# Experimental Study on Microfluidic Mixing with Different Zigzag Angles

**DOI:** 10.3390/mi10090583

**Published:** 2019-08-31

**Authors:** Chia-Hung Dylan Tsai, Xin-Yu Lin

**Affiliations:** Department of Mechanical Engineering, National Chiao Tung University, Hsinchu 30010, Taiwan

**Keywords:** microfluidic mixing, diffusion, advection, zigzag angle

## Abstract

This paper presents experimental investigations of passive mixing in a microfluidic channel with different zigzag angles. Zigzag channel is commonly used for microfluidic mixing because it does not need an additional control unit and can be easily implemented in a lab-on-a-chip system. In this work, microfluidic channels with six different zigzag angles, from θ = 0° to θ = 75°, are tested under ten different flow rates corresponding to Reynolds number from 0.309 to 309. Two colored liquids are mixed with the zigzag channels and mixing performance is evaluated based on the color of the pixels on the region of interest from captured images. According to the results, we found that the mixing performance is almost independent of the zigzag angle in the low-speed regime where its Reynolds number is less than 4. The mixing became very much depending on the zigzag angle in the high-speed regime where its Reynolds number is greater than 100. Microfluidic mixing is needed for Lab-on-a-chip applications in both low flow speed, such as medium perfusion for cell culture, and high flow speed, such as high-speed sensing on a point-of-care device. This work is aimed to provide practical information on zigzag mixing for chip design and applications.

## 1. Introduction

Microfluidic mixing is important for improving uniformity and consistency on lab-on-a-chip (LOC) systems. While mixing can be an easy task in macro-scale environment, low Reynolds number makes mixing a challenging task in microfluidic environment. It is because that the low Reynolds number results in a laminar flow where mixing could only happen at the boundaries of fluidic layers by spontaneous diffusion. While microfluidic platform becomes more and more popular for studies in biological cells [1,2,3,4], a simple and effective mixing for such applications is on demand. Passive mixing with a zigzag channel is one of the most popular methods because it is convenient, easy-to-fabricate and control-free. This work aims to reveal the performance of microfluidic mixing with different zigzag angles through experiments for the effective use of a zigzag channel in a LOC system.

Different methods for assisting microfluidic mixing have been proposed and discussed in literature, and they can be categorized into active and passive approaches [5]. Active approaches mostly generate vortices by external actuation to enhance the mixing. For example, Cheaib et al. presented a numerical study of mixing with pulsating flows in microflows, and found that the optimal frequency depends on the pulsation amplitude and channel width [6]. Shang et al. generated vortices with piezoelectric actuators in a microfluidic chamber for improving on-chip mixing [7]. Zhou et al. designed a magnetically functionalized flexible micropillar arrays for rapid microfluidic mixing [8]. Wang et al. utilized nonlinear electrokinetic vortex for mixing [9]. Chen and Cho combined active and passive mixing techniques and demonstrated improved mixing with a greater wave amplitude or extended length of the wavy-wall section [10]. Kim et al. induced chaotic flow for mixing by periodic perturbation of velocity fields with barriers along the top surface of the channel [11]. Takayama et al. enhanced mixing by a high-speed actuation and adding bio-compatible surfactant to the driving fluid [12].

Passive approaches, on the other hand, are the methods enhancing the mixing with different channel structure, and is generally more convenient than active ones because control of actuation is not necessary [13]. Studies on the structure of channel walls, such as T-shaped micromixer (TM), slanted groove micromixer (SGM), staggered herringbone micromixer (SHM), barrier-embedded chaotic micromixer (BEM), circulation disturbance micromixer (CDM), are the majority of the study in the last few decades. For example, Stroock et al. demonstrated the generation of flows on cross-sections of a SHM mixer using confocal micrographs [14]. Yang et al. showed that CDM performed a better mixing than TM, SGM, BEM and SHM within the Reynolds number between 10^−2^ and 10^2^ [15]. There are also passive approaches using single-layer planar design, such as the zigzag channels in this work. For example, Lin et al. enhanced mixing by J-shaped baffles and showed that the percentage of mixing increased as the number of baffles increased [16]. Chung et al. utilized self-circulation of the fluid in a circular chamber and showed enhanced mixing performance [17]. Fu et al. demonstrated that a double-heart chamber design can effectively enhance the mixing [18].

Zigzag channel is a kind of passive mixing with planar structure, and is particularly convenient for microfluidic mixing because it is control-free and easy to fabricate. Different studies of microfluidic mixing with a zigzag channel have been reported [19,20,21,22,23]. For example, Mengeaud et al. provided a theoretical study on mixing with a zigzag channel using Navier-Stokes equation and diffusion-convection equation in the steady state form for different zigzag ratios of channel width and periodic step distance [24]. Ren et al. developed a numerical model accounting for the interactions of throughflow, crossflow and material dispersion by diffusion and convection in a rotational platforms, and the simulation results were found match to the experimental results at two selected rotation speeds [25]. To the best of the authors’ knowledge, this work first provides experimental evidence showing the relationship between the zigzag angle and mixing performance in a stationary zigzag channel. Microfluidic mixing with six different zigzag angles is investigated with a 1000-fold span of Reynolds number from 0.309 to 309. An image-based method is utilized for evaluating the performance of the mixing.

The rest of this paper is organized as follows: The experimental method is explained in Section 2. The results are presented in Section 3. Discussions on mixing in a zigzag channel are presented in Section 4. Finally, the concluding remarks are summarized in Section 5.

## 2. Method

### 2.1. Overview

**Figure 1:**
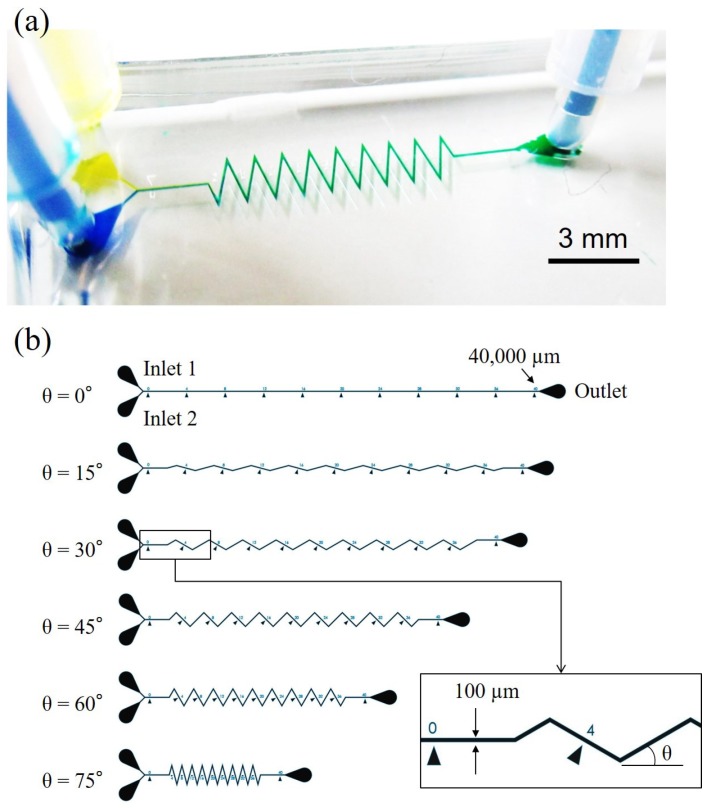
An overview of a zigzag mixing channel and zigzag angles. (**a**) A photo of the microfluidic chip in experiments. (**b**) Designs of microfluidic mixing channel with six different zigzag angles.

Figure 1 shows an overview of the work while Figure 1a,b are a photo of the microfluidic chip and the designs of six different zigzag angles. The test channel consists of two inlets and one outlet, as shown in Figure 1a. Two different color liquids, yellow and blue, are injected into the channel from the two inlets, and they are drained out from the outlet after passing through the zigzag channel. The mixing performance can be evaluated according to the color of the pixels on a region of interest (ROI). Zigzag angle and flow rate are two parameters particularly important for a LOC system and are studied in this work. For example, a user of LOC system is not necessarily familiar with control systems but they can use a commercial syringe pump to perform an effective mixing if a preferable flow rate is given.

### 2.2. Experimental System

**Figure 2:**
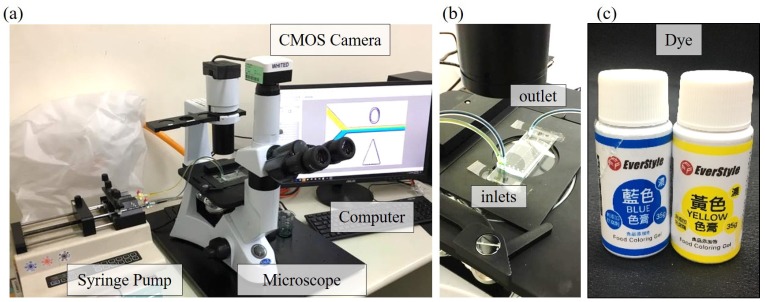
The experimental setup. (**a**) System overview. (**b**) Connections of inlets and outlet on the chip. (**c**) Color dyes for the mixing experiments.

Figure 2a shows a photo of the experimental setup, where it consists of a syringe pump (Diagnostic & Research Instrument Co., Ltd., Taoyuan, Taiwan), a microscope (SAGE Vision Co., Ltd., New Taipei, Taiwan), a complementary metal-oxide semiconductor (CMOS) color camera (lWhited Ltd., Taipei, Taiwan), a computer and a microfluidic chip. The syringe pump can simultaneously push two syringes at the same time which is convenient for providing the same flow rate at two inlets in the mixing tests. The color camera is for recording images through the microscope. The performance of mixing is analyzed based on the images with its color components. Two inlets and one outlet are on the microfluidic chip, as shown in Figure 2b. Yellow and blue color liquids made of food coloring gels (EverStyle Co., New Taipei, Taiwan), as shown in Figure 2c, are prepared at the water-gel ratio of 10:1, and both their densities are measured as 1 g/cm^3^. The liquids are pushed into the channel from the two inlets at the same flow rate using the syringe pump, and the combined liquid is flowed out of the chip from the outlet.

### 2.3. Dimensions and Fabrication of Microfluidic Channels

**Figure 3:**
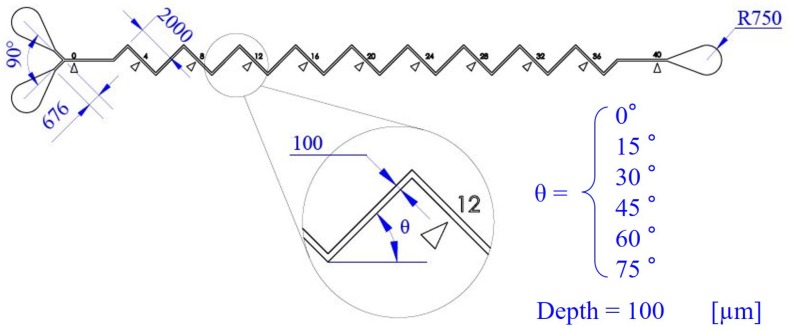
The dimensions of the zigzag channels used in experiments. Channels with six different zigzag angles θ are fabricated while all the other dimensions are kept the same.

Figure 3 shows the dimensions of different zigzag channels and the definition of the zigzag angle θ. The width, depth and total length of the six channels are 100 µm, 100 µm and 40,000 µm, respectively. The zigzag angle is defined as the angle between the winding channel and the horizontal line, as illustrated in Figure 3. Microfluidic channels with six different zigzag angles are fabricated and the angles are, from low to high, 0°, 15°, 30°, 45°, 60° and 75°. There are numerical marks beside the channel as shown in Figure 3, and they are for identifying the location of mixing during the experiments.

The Microfluidic channels are fabricated from a mold which is made with standard photolithography process [26]. For making the mold, a silicon wafer is first coated with a 100 µm thick photoresist SU8-3050 (MicroChem Corp., Westborough, MA, USA) by a spin coater. Two-step coating process is adapted to improve the uniformity of the thickness.The photoresist is first coated at 500 revolutions per minutes (rpm) for 20 s for spreading out the photoresist over the silicon substrate, and is followed by 1400 rpm for 30 s for controlling the height of the coat. The designs of the channels with different zigzag angels, as shown in Figure 1b, are printed on a plastic mask, and are patterned on the coated silicon wafer using the mask and an alignment device. The fabrication of the mold is completed after the silicon wafer is developed. A mixture of polydimethylsiloxane (PDMS) (DC184, Dow Corning, Midland, TX, USA) and its curing agent is prepared at the PDMS-agent ratio of 10:1. The mixture is poured onto the mold in a disposable disk, and is processed by a 20-min de-gasing in a vacuum chamber and 40-min baking in a 95 °C. After the PDMS is cured, the chip is removed from the mold and two inlets and one outlet are punched using a biopsy puncher. Finally, the PDMS chip is bonded to a glass substrate using argon plasma. Low-temperature plasma is generated from an atmospheric pressure plasma jet and is evenly applied on the surface of both the PDMS chip and glass substrate for few seconds before stacking two pieces together for the bond. The fabrication of the chip is completed after the bond.

### 2.4. Image-based Evaluation for Mixing Performance

The evaluation for the mixing performance is based on the color of the pixels on a ROI of captured images. The ROI for the mixing analysis is set as a 200 µm long and 70 µm wide region inside the channel. The choice of 70 µm instead of the full width of 100 µm of the channel is to avoid unstable color due to the shadow from the channel walls. The mixing index of the ROI is defined as

(1) $$M=\left[1-\frac{1}{N}\sum_{i=1}^{N}\sqrt{{\left(\frac{\alpha_{i}-\alpha_{ref}}{\alpha_{ref}}\right)}^{2}}\right]\times 100\%$$

where *M*, *N*, $\alpha_{i}$ and $\alpha_{ref}$ are the mixing index, the total number of pixels in the analyzed region, the mixing level of *i*th pixel and the mixing level of a perfect mix, respectively. The mixing index *M* is ranged from 0% to 100% where 0% and 100% represent the worst and the best mixing, respectively. For example, if two fluids are completely separated in two layers, which is the worst mixing, the mixing level $\alpha_{i}$ of the pixels from a captured image would be either 100% or 0% and the level of the perfect mix $\alpha_{ref}$ would be 50%. Thus, Equation (1) can be written as

$$\begin{array}{ccc} M & = & \left[1-\frac{1}{N}\sum_{i=1}^{N}1\right]\times 100\%\\ & = & \left[1-\frac{N}{N}\right]\times 100\%\\ & = & 0\% \end{array}$$

On the other hand, if the mixing is to be perfect, the mixing level $\alpha_{i}$ of the pixels would be 50% for all the pixels. The mixing index *M* can be obtained as

$$\begin{array}{ccc} M & = & \left[1-\frac{1}{N}\sum_{i=1}^{N}0\right]\times 100\%\\ & = & \left[1-\frac{0}{N}\right]\times 100\%\\ & = & 100\% \end{array}$$

**Figure 4:**
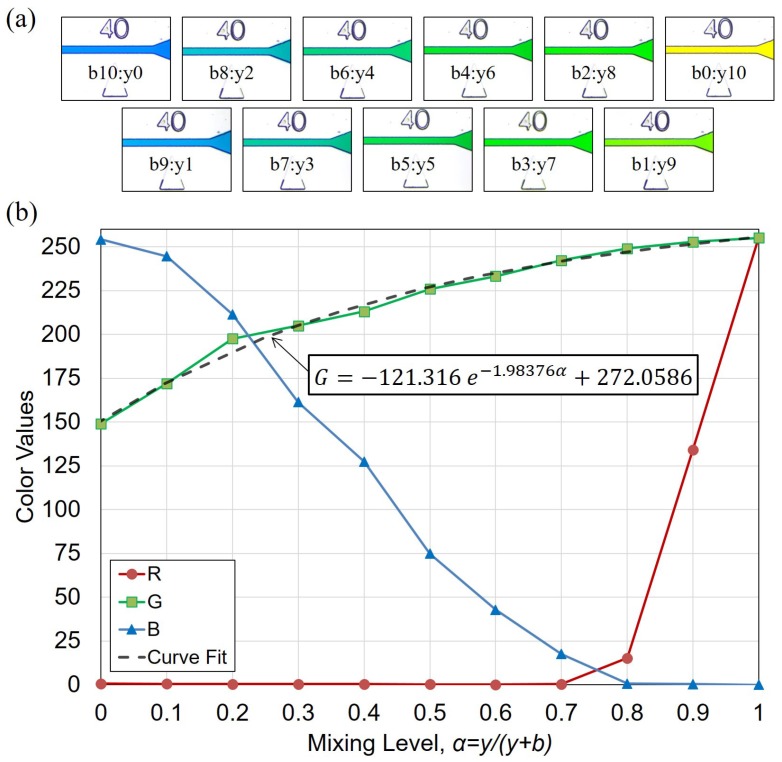
The calibration of color components and mixing ratio. (**a**) Images of reference colors in the channel. (**b**) R, G, B components at different mixing levels α.

The mixing level α is calculated based on the reference colors, which are experimentally obtained before the experiments. The reference colors are made of blue and yellow dyes at eleven different ratios in a beaker before injecting into the channel. The images of the reference color in the channel are shown in Figure 4a. The labels in Figure 4a indicate the ratio of blue and yellow dyes in the mixture. The mixing level α is defined as the concentration of yellow dye and can be written as α=y/(y+b) where *y* and *b* are the portion of yellow and blue dyes, respectively. For example, “b3:y7” in Figure 4a indicates the blue-yellow ratio of 3:7 and α of 0.7.

Figure 4b shows the evolution of color components red (R), green (G) and blue (B) with respect to different mixing levels α. It is found that G component has a 1-to-1 mapping with the mixing level α while R and B do not. Curve fitting is applied to the G-α relation with an exponential equation as

(2) $$G=-121.316e^{-1.98376\alpha }+272.0586$$

The fir curve is shown as the dashed line in Figure 4b, and $R^{2}>0.99$ of the fit shows a good fit. The mixing level α of a given color can then be determined based on its G component with Equation (2).

### 2.5. Calculation of Reynolds Number, Re

Reynolds number is a dimensionless quantity for describing a fluidic environment. It is the ratio between inertial force and viscous force, and can be written in the form [27]

(3) $$Re=\frac{\rho VL}{\mu }$$

where ρ, *V*, *L*, μ are the density of the fluid, the flow speed in the channel, the characteristic length of the channel, and dynamic viscosity of the fluid, respectively. The density and dynamic viscosity of the fluid in this paper are set as ρ = 1 × 10^3^ kg/m^3^ and μ = 1.00 mPa·s as those of water at 20 °C.

For the characteristic length, *L*, general formula for *L* is the volume of the system divided by its surface, or in hydraulic case it often calculated as the area of the cross-section divided by its wetted perimeter

(4) $$L=\frac{4A}{p}$$

where *A* and *p* are the cross-sectional area of the channel and the perimeter of the cross-section, respectively. In this case, we have *A*= 1 × 10^−8^ m^2^ and *p* = 4 × 10^−2^ m. Therefore, the characteristic length in Re calculation *L* = 1 × 10^−4^ m.

For the flow speed, *V*, it is measured from the actual speed of tracked microbeads using a high-speed camera (PCO Co. Ltd., Kelheim, Germany), instead of averaging the flow rate by the cross-sectional area. The consideration of using the measured speed is to avoid underestimation of the flow speed due to unknown velocity profile on the cross-section of the channel. The speed of 3.08 mm/s was obtained from the experiment at the lowest flow rate of 0.001 mL/min. The average of the flow speed over the cross-section of 100 × 100 µm^2^ is 1.67 mm/s, which is only about half of the measured speed. The speeds of different flow rates are proportionally determined based on the measured speed at the lowest flow rate. For example, the speed at the flow rate of 0.1 mL/min is determined as 308 mm/s. Thus, the flow rates tested in this paper results in a 1000-fold span of Reynolds number from Re = 0.308 to Re = 308.

## 3. Results

There are two parts of the experimental results. First, the mixing at different locations along the zigzag channel of θ = 45° is investigated. The purpose is for understanding how the mixing is progressed along the channel under different flow rates. Three different orders of flow rates, 0.01 mL/min, 0.1 mL/min and 1 mL/min, are tested, and images at specified locations are captured for analysis. The second part of the experiments is on all six zigzag angles at ten different flow rates ranging from 0.001 mL/min to 1 mL/min. The images of the mixing under different zigzag angles and flow rates are captured and analyzed using Equation (1).

### 3.1. Mixing at Different Locations along the Channel

**Figure 5:**
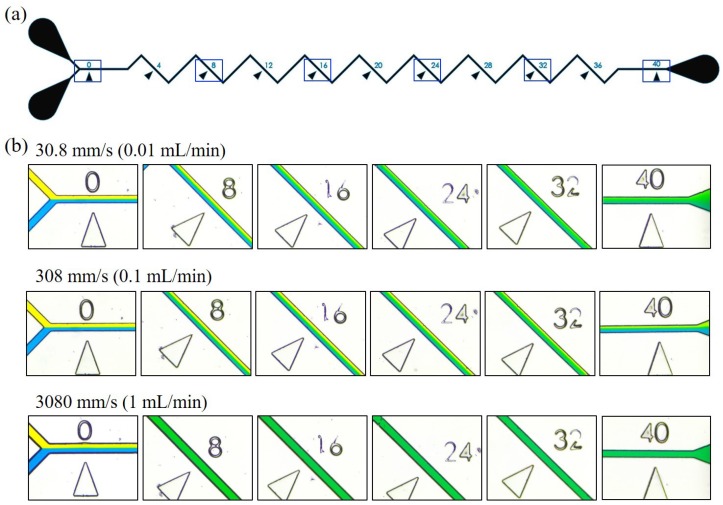
Experimental results of mixing performance at different locations in a zigzag channel. (**a**) The boxes on the channel show the locations of observation. (**b**) The experimental results of mixing at the locations under three different flow rates.

Figure 5 shows the results of mixing at different locations along the zigzag channel. There are six observation windows at 0 mm, 8 mm, 16 mm, 24 mm, 32 mm and 40 mm, as the highlighted blue boxes shown in Figure 5a. The images of actual mixing at different observation windows are captured and shown in Figure 5b. The three rows of Figure 5b, from the top to the bottom, are the results of flow rates at 0.01 mL/min, 0.1 mL/min and 1 mL/min. The same initial condition for all the three different flow rates can be confirmed at the locations of 0, where the yellow and blue liquids are in the layers of equal size. The speed labeled on the upper-left corner of each row is the measured speed corresponding to the flow rates, and they are 30.8 mm/s, 308 mm/s and 3080 mm/s, respectively.

According to the results in Figure 5b, it is found that the mixing is not necessarily improved with the increase of the flow rate. Among three flow rates in Figure 5b, the middle flow rate of 0.1 mL/min shows the worst mixing that color layers can still be seen from the captured image at 40 mm. For the low flow rate of 0.01 mL/min, the two colors are gradually mixed as the progressively increasing width of the green band at the middle of the channel. The green band covered the whole channel at the location of 40 mm, and it indicates a good mix even in such a low flow rate. When it came to the highest flow rate of 1 mL/min, the mixing is immediately done at the location of 8 mm where channel is all covered by green color.

### 3.2. Mixing with Different Zigzag Angles at Different Flow Rates

**Figure 6:**
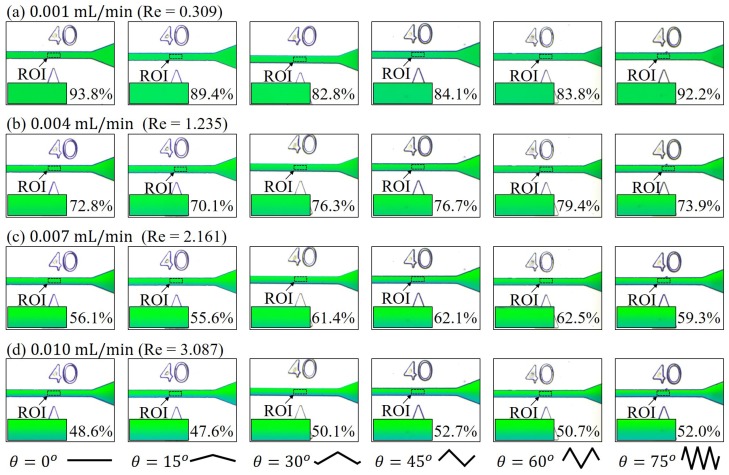
Experimental results of mixing with six different zigzag angles at low flow rates. (**a**) 0.001 mL/min. (**b**) 0.004 mL/min. (**c**) 0.007 mL/min. (**d**) 0.01 mL/min.

Experiments with all six zigzag angles are conducted under three sets of flow rates in different orders, and are named as low, medium and high flow rates in the results. Figure 6 shows the experimental results of the first set of low flow rates, where Figure 6a–d are the results at 0.001 mL/min, 0.004 mL/min, 0.007 mL/min and 0.010 mL/min, respectively. The columns in Figure 6, from the left to the right, show the mixing results at the location of 40 mm with the zigzag angles of θ = 0°, 15°, 30°, 45°, 60°, 75°.

A trend on mixing and the flow rates can be seen from Figure 6a to d that the separation of layers became more and more clear with the increase of the flow rate in all six zigzag angles. For example, the enlarged ROIs at the flow rates of 0.001 mL/min in Figure 6a seem to have a fairly well mix and the calculated *M* from Equation (1), are ranged from 82.8% to 93.8% among the six different zigzag angles. The *M* values for the ones at 0.010 mL/min in Figure 6d, whose flow rates are 10 times faster than the ones in Figure 6a, are ranged from 47.6% to 52.7%. The difference of mixing performance between the flow rates of 0.001 mL/min and 0.010 mL/min is significant and it indicates that, in such a low-speed regime, a higher flow rate would result in a worse performance of mixing. On the other hand, there is no clear difference between the mixing results among the six different zigzag angles, which demonstrate that the zigzag angle is not critical in the regime of low flow rates.

**Figure 7:**
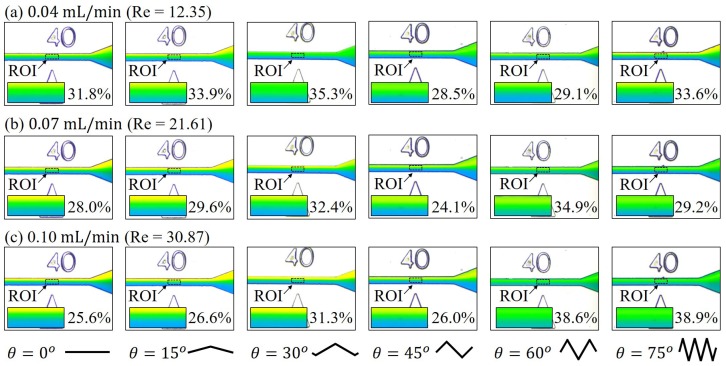
Experimental results of mixing with six different zigzag angles at medium flow rates. (**a**) 0.04 mL/min. (**b**) 0.07 mL/min. (**c**) 0.10 mL/min.

Figure 7 shows the results of mixing at medium flow rates, which are an order greater than those in Figure 6. The flow rates are from 0.04 mL/min to 0.10 mL/min. In the regime of medium flow rates, we can find that different zigzag angles would lead to different mixing results, unlike the results in the regime of low flow rates in Figure 6 that mixing performance is nearly independent of the zigzag angles. The effect of zigzag angle becomes more pronouncing with the increase of the flow rate. For example, the width of green band at the flow rate of 0.10 mL/min in Figure 7c becomes wider with the increase of the zigzag angle. The *M* calculated by Equeation (1) for the angle of θ = 0° to 75° at the flow rate of 0.10 mL/min are 25.6%, 26.6%, 31.3%, 26.0%, 38.6% and 38.9%, respectively. The mixing performance is progressively improved with the increase of the zigzag angles. The mixing indices with θ = 45° are found lower than the indices of nearby zigzag angles, such as θ = 30° and θ = 60° in Figure 7. It could be an experimental error due to micro-scale defect in the channel while it may also be another phenomenon which is required further investigation.

**Figure 8:**
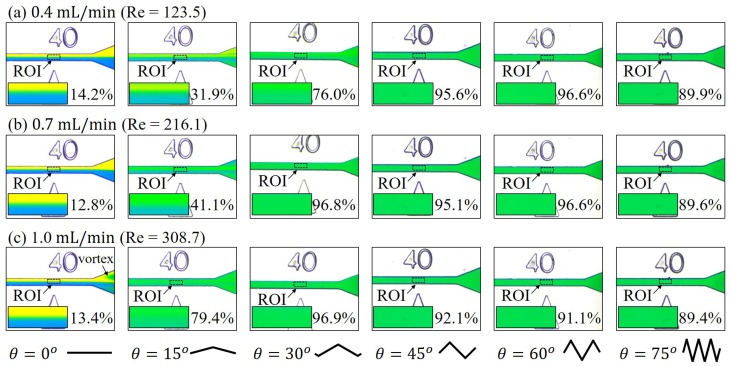
Experimental results of mixing with six different zigzag angles at high flow rates. (**a**) 0.4 mL/min. (**b**) 0.7 mL/min. (**c**) 1.0 mL/min.

Figure 8 shows the results of mixing at high flow rates, which are ranging from 0.4 mL/min to 1.0 mL/min. The flow rates in Figure 8 are another order greater than the ones in Figure 7. In the regime of high speed, it is very clear that a greater zigzag angle leads to a better mix while the angle of zero θ=0 gives the worst mix. For example, the results of the flow rate at 0.4 mL/min in Figure 8a shows a progressive improvement of mixing from θ = 0° to θ = 45° while the mixing seems to be saturated from θ = 45° to θ = 75°. There are observable vortices in the triangular area close to the outlet for the channel of θ = 0° at the flow rate of 1.0 mL/min.

**Figure 9:**
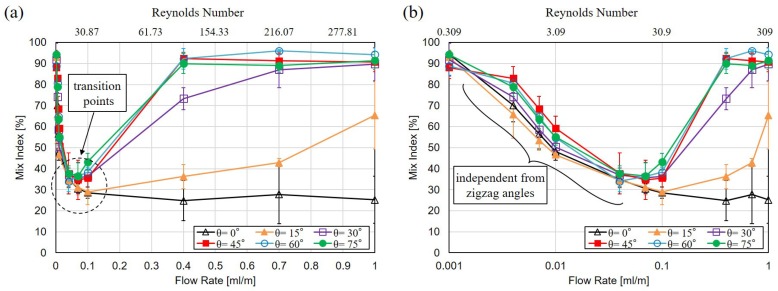
Experimental results of mixing performance under 6 different zigzag angles, including θ = 0°, 15°, 30°, 45°, 60° and 75°. The marks and error bars represent the average values and standard deviations among three independent tests. (**a**) The analyzed results of mixing index with different zigzag angles at different flow rates. (**b**) The chart in logarithmic scale for the flow rate.

Figure 9 summarizes the analyzed results by Equation (1) for the mixing performance with different zigzag angles at the flow rates from 0.001 mL/min to 1.0 mL/min. Figure 9a,b are the plot of the same results in linear scale and non-linear logarithmic scale, respectively. Each test has been repeated 3 times. The data points in Figure 9 represent the average value among all the results while the error bars are the standard deviations.

According to the results in Figure 9, three interesting phenomena are observed. First, there is a transition point of mixing performance for all zigzag channels, except the straight channel with θ=0 as the black solid line with hollow triangle marks in Figure 9. The mixing performance is gradually decreased from a low flow rate to a medium flow rate. After reaching a transition point, the mixing is improved from the medium flow rate to a high flow rate. The transition point is found consistently around the regime of Re=15 to Re=50 for the zigzag angles greater than zero.

The second observation is that the mixing performance is nearly the same among different zigzag angles in the regime of low flow rates where Re<30, as the analyzed results shown in Figure 9b. There are still differences between the analyzed results from different zigzag angles, but no consistent trend of mixing is found, so the difference may due to the errors in experiments. This result shows that the zigzag angle is independent of the mixing performance when the Reynolds number is in the order of 30 or less. We would like to note that all the channel are kept with the same dimensions of their cross-sections as well as the total length, and the only difference among different channel is the zigzag angles.

The third observation is that the mixing performance is very different when the flow rate is greater than the transition points. For the zigzag angle of 0°, the mixing is continuously getting worse simply due to a shortened mixing time, which is a result of the high flow rates. For the zigzag angle of 15°, the mixing is slowly improved with the increase of flow rate and reached the best mixing at the highest flow rate of 1 mL/min, as the solid line with filled triangles in Figure 9. For the zigzag angles of 30°, the mixing is improved with the flow rate after passing the transition point, and the mixing is saturated when the flow rate is beyond 0.7 mL/min, which is corresponding to Re=216. For the zigzag angles greater than 45°, the mixing performance in Figure 9 are rapidly improved with flow rates as the convex curves in Figure 9. According to the results, the mixing reaches saturation when the flow rate is beyond 0.4 mL/min, which is corresponding to Re=123.2.

## 4. Discussion

### 4.1. Mixing of Zigzag Angles in Different Speed Regimes

The low-speed results in Figure 6 are reasonable considering their Reynolds number are in the range between Re=0.3 to Re=3.0. In such low Reynolds numbers, the flows are expected to be laminar and layers of fluids would be parallel to the channel walls. Therefore, the mixing would be governed by the laws of diffusion. For example, Fick’s first law of diffusion explains the evolution of non-uniform concentration in a steady-state, and it gives

(5) $$J=-D\frac{\partial c}{\partial x}$$

where *J*, *D*, *c*, *x* are the diffusion flux, the diffusion coefficient, the concentration, and the position, respectively. It shows that the magnitude of flux, which indicates the flow of substance per unit cross-section per unit time, is proportional to the gradient of concentration and is always directed from a high-concentration region to a low-concentration region. The diffusion can be considered as steady-state for the low Reynolds number flows in Figure 6 because the flow is laminar and there is no significant flow along the direction perpendicular to the flow direction, except the spontaneous diffusion. In other words, the mixing could solely depend on the time of diffusion in the regime of low flow rates. A lower flow rate results in a longer time of diffusion in a fixed channel length, so that the mixing results with a slower flow rate are better than the mixing with a greater flow rate in this regime. In addition, no clear difference of mixing between different zigzag angles in such a low-speed regime.

The mid-speed results in Figure 7 can be interpreted as advective flux joined the governed equation of the mix while there is only diffusive flux in the low-speed regime. The advective flux becomes more dominant with the increase of the flow rate while diffusive flux remains almost constant since it only depends on the diffusion coefficient and the gradient of concentration of the fluids. Therefore, the dependency of the zigzag angle becomes more obvious in the medium flow rates than in the low flow rates.

The Reynolds numbers in the high-speed regime in Figure 8 are between 123.5 and 308.7, where microfluidic vortices can be generated around this regime as previously reported in literature [28,29]. The swirling motion of vortices is commonly used to enhance mixing by increasing contact between the two colors. The straight channel with θ=0 is a special case that the inertia of fluidic molecules does not vary, and thus the mixing is not enhanced with the speed. All mixing channels with Zigzag angles greater than zero demonstrate an enhanced mixing compared with low-speed and mid-speed flow rates. For the angles greater than 30°, a saturation of mixing can be found from the trend of the curves in Figure 9.

### 4.2. Experimental Errors in Flow Control and Channel Dimensions

As a nature of experimental study, errors could come from various practical situations which are sometimes neglected during theoretical investigations. Two experimental errors, including flow control and channel dimensions, are discussed here. In terms of flow control, syringe pumps have been an useful tool for microfluidic platforms. However, fluctuations during the infusion is unavoidable as most of the pumps are driven by a stepper motor with finite step size Li et al. reported a visualization of such a fluctuation at different flow rates [30]. As a result, pulsating flow is possible to be generated during the experiments and is likely improve the mixing.

**Figure 10:**
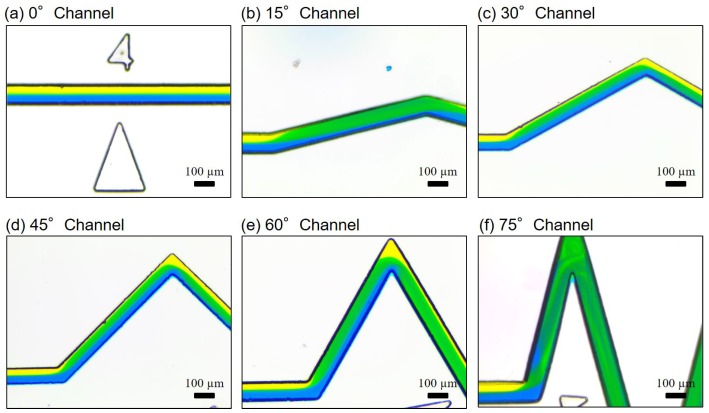
Experimental results of spiral-like advection in different zigzag channels at the flow rate of 1.0 mL/min (Re=308.7). (**a**) θ = 0°. (**b**) θ = 15°. (**c**) θ = 30°. (**d**) θ = 45°. (**e**) θ = 60°. (**f**) θ = 75°.

On the other hand, the uniformity of the thickness of the mold is also an important factor for the channel dimension. According to experimental measurement, the maximum difference of height in a channel can be 8 µm for the channel with cross section of 100 µm × 100 µm. Such a thickness error would lead to the variation of Reynolds number around 3.8% according to Equations (3) and (4). Dilation of the channel can be observed from Figure 10 at high flow rates, and could be another factor affecting the mixing of a microfluidic zigzag channel. The channel width may be dilated due to the high pressure from the high flow rate and the increased resistance of zigzag channels. The dilation would benefit the mixing in experiments because the Reynolds number would become bigger with an increased characteristic dimension according to Equation (4).

### 4.3. Spiral-Like Advection in Different Zigzag Angles

Figure 10 shows captured photos of advective flows with different zigzag angles at the high flow rate of 1.0 mL/min. The Reynolds number of the flow rate is calculated as 308.7 based on Equation (3) with the parameters in Section 2.5. The spiral-like rotation of two layers of fluids can be found from all the channels, except the straight channel with θ = 0°. This explains why the mixing index of the straight channel is the only one getting worse in the high flow rates in Figure 9a. In addition, an interesting phenomenon in Figure 10c–e is that the yellow color is once disappeared in the zigzag channel but is later shown up again at the turning corner. This phenomenon indicates that a well-mixed color, green in this case, may result from overlapping of different colors layers, blue and yellow here, from the viewpoint of the camera, instead of an actual mix.

While diffusion is the fundamental mechanism of mixing and is driven by the gradient of concentration as formulated in Equation (5), advection creates jumbled up fluidic layers of different concentrations, which speed up the progress of diffusion due to rugged gradients of concentration. Advection is believed to be the main reason of good mixing in a channel with a greater zigzag channel at high flow rates. Student T test were applied to the mixing indices of low flow rates (0.001 mL/min–0.007 mL/min) between zigzag angle of 0° and other zigzag angles. The *p* values for the angles of 15°, 30°, 45°, 60° and 75° are 43%, 47%, 32%, 38% and 37%, respectively. It indicates that there is no significant difference of mixing between different zigzag angles at low flow rates.

## 5. Conclusions

Microfluidic mixing on zigzag channels with six different zigzag angles is experimentally investigated in this paper. Experiments for a 1000-fold span of Reynolds number, from 0.309 to 309, have been conducted. According to the experimental results, we found that there is a clear transition between diffusion-based and advection-based mixing for the Reynolds number around 30. The mixing performance is the worst at the transition. The mixing is improved with either an increased or a reduced Reynolds number from the transition. It is also found that the mixing performance is independent of the zigzag angle in low-speed regime where the Reynolds number is less than 4. That means if a LOC system is designed to operate in a low-speed perfusion, a zigzag angle design for mixing is unnecessary. On the other hand, the zigzag angle of 45°, or greater, effectively improves the mixing in the high-speed regime where Reynolds number is greater than 100.

## Abbreviations

The following abbreviations are used in this manuscript:

LOC	lab on a chip
ROI	region of interest
